# Clinical, Imaging, and Serum Biomarker Predictors of Malignant Cerebral Infarction

**DOI:** 10.3390/jcdd12100392

**Published:** 2025-10-04

**Authors:** Alejandro Rodríguez-Vázquez, Salvatore Rudilosso, Antonio Doncel-Moriano, Andrea Cabero-Arnold, Carlos Laredo, Darío Ramis, David Moraleja, Mònica Serrano, Yolanda González-Romero, Arturo Renú, Inés Bartolomé-Arenas, Irene Rosa-Batlle, Guillem Dolz, Ramón Torné, Martha Vargas, Xabier Urra, Ángel Chamorro

**Affiliations:** 1 Faculty of Medicine, Universitat de Barcelona, 170 Villarroel, 08036 Barcelona, Spain; alrodriguez@clinic.cat (A.R.-V.);; 2 Department of Neurology, Hospital Clínic de Barcelona, 08036 Barcelona, Spain; srudilos@clinic.cat (S.R.);; 3 Fundació de Recerca Clínic Barcelona—Institut d’Investigacions Biomèdiques August Pi i Sunyer (FRCB-IDIBAPS), 08036 Barcelona, Spain; 4 Department of Radiology, Hospital Clínic de Barcelona, 08036 Barcelona, Spain; 5 Department of Neurosurgery, Hospital Clínic de Barcelona, 08036 Barcelona, Spain

**Keywords:** large vessel occlusion, malignant cerebral infarction, cerebral edema, CT perfusion, biomarkers, predictive modelling

## Abstract

Malignant cerebral infarction (MCI) is rare but often fatal. Early identification helps guide monitoring and decompressive surgery. This study evaluated whether serum biomarkers add predictive value beyond clinical and imaging data in severe stroke patients with anterior circulation large vessel occlusion (LVO). In this prospective study, 73 acute severe LVO stroke patients underwent whole-brain CT perfusion (CTP) with rCBV-based core measurement at admission and follow-up MRI at 24 ± 12 h for infarct and edema volume assessment. Serum biomarkers (s100b, NSE, VEGF, ICAM1) were sampled a median of 20.5 h after baseline imaging. Logistic regression models predicted MCI using baseline variables (NIHSS, ASPECTS, rCBV < 30%), adding treatment data (rtPA, mTICI, NIHSS posttreatment), and adding serum biomarkers. Performance was assessed by AUC, accuracy, F1, and cross-validated R^2^. MCI occurred in 18/73 (24%) patients. Baseline models showed an AUC of 0.72; adding treatment improved the AUC to 0.88. Biomarkers slightly increased the AUC (0.90) but did not improve F1. Higher s100b was associated with more severe injury but did not enhance the prediction of MCI. Models with baseline imaging and treatment best explained infarct (R^2^ ≈ 0.27) and edema (R^2^ ≈ 0.58). In conclusion, admission severity, CTP, and early treatment response are the main predictors of MCI and aid early risk stratification of patients. Despite their pathophysiologic relevance, serum biomarkers do not add substantial predictive value.

## 1. Introduction

Ischemic stroke is currently one of the leading causes of death and disability worldwide [1,2]. The term “malignant cerebral infarction” (MCI) was introduced by Hacke et al. in 1996, referring to extensive ischemia involving virtually the entire territory irrigated by the middle cerebral artery [3]. These patients presented with severe hemispheric syndromes upon arrival, followed by symptoms of intracranial hypertension within a time frame of two to five days. Serial neuroimaging studies in such patients revealed a gradual increase in cerebral edema that led to herniation and compression of vital structures in 78% of cases. The prognosis was poor, with a mortality rate of nearly 80% and a high burden of sequelae in survivors [4].

Approximately 5 to 10% of ischemic strokes follow this malignant course, which is also more likely in young patients, as the absence of cerebral atrophy prevents accommodation to the increase in volume secondary to edema [5,6]. Currently, there is no effective medical therapy for MCI [7,8,9]. The only treatment that has improved survival is decompressive hemicraniectomy, although with a moderate or severe degree of residual disability [10,11,12,13]. Timing is crucial when deciding on surgical intervention, so early diagnosis is helpful to streamline decision-making [14]. Classically, predictive models for diagnosing malignant cerebral infarction (MCI) rely on magnetic resonance imaging (MRI), with infarcts over 145 cc being at particular high risk of developing MCI (15). However, MRI may not be readily available in many instances, particularly for patients in a poor clinical situation [15]. CT-perfusion (CTP) is a more commonly available tool that could be useful in identifying early patients at risk of developing malignant cerebral edema [16]. A cerebral infarction that occupies two-thirds of the territory of the middle cerebral artery, or a smaller volume but with involvement of the basal ganglia or other vascular territories, has a high risk of developing MCI [17].

The evaluation of the neuroinflammatory response based on the systemic expression of different leukocyte subtypes has been linked to the functional outcome in acute ischemic stroke [18]. Furthermore, previous research studied the effect of various systemic biomarkers, including s100b, metalloproteases, and interleukins, on the development of MCI [19,20,21]. More recent studies have investigated the involvement of markers such as kisspeptin [22,23] and microRNAs [24,25] in cerebrovascular damage and the development of cerebral edema. However, the clinical utility of serum biomarkers is not clear, and they are not systematically used for predicting MCI in routine practice.

Following the publication of clinical trials demonstrating the efficacy of mechanical thrombectomy in extensive cerebral infarctions [26,27,28,29,30], the early identification of patients at high risk of developing MCI has become more relevant. In this study, we aim to evaluate the usefulness of serum determination of a series of circulating biomarkers potentially involved in the pathophysiology of cerebral ischemia, such as s100b [31,32], neuron-specific enolase (NSE) [33,34], Vascular Endothelial Growth Factor (VEGF) [35,36], and Intercellular Adhesion Molecule 1 (ICAM-1) [37]. In particular, we investigated their role in the development of MCI and their value compared to clinical and neuroimaging information.

## 2. Materials and Methods

**Patient selection**

**Figure 1:**
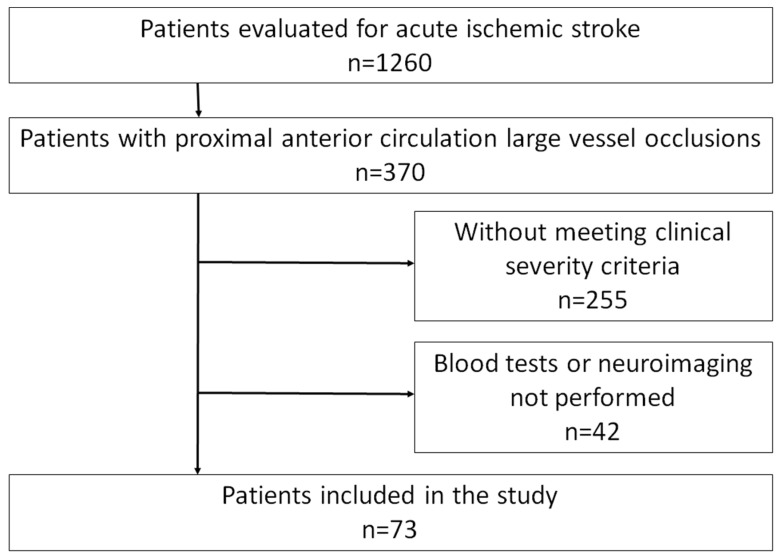
Flow chart describing screening, exclusions, and the final cohort.

We prospectively evaluated 1260 consecutive acute ischemic stroke patients, of whom 370 had proximal anterior circulation large vessel occlusion (LVO). After applying selection criteria, the analytical cohort consisted of 73 patients at increased risk for malignant evolution (see Figure 1). The following were the inclusion criteria:

1. Proximal LVO involving the carotid artery or M1 segment of the middle cerebral artery (MCA).
2. Acute hemispheric syndrome with severe symptoms (National Institute of Health Stroke Scale (NIHSS) > 15 for dominant hemisphere or NIHSS > 13 for non-dominant hemisphere) at admission.
3. Lack of clinical improvement (more than four points in the NIHSS) after treatment or in the first 24 h after admission.
4. Admission multimodal CT protocol, including non-contrast CT (NCCT), CT angiography (CTA), and CTP.

All patients had follow-up neuroimaging (CT and MRI) conducted within 24 +/− 12 h of admission, serial blood tests performed on admission and in the first 24 h after the initial neuroimaging, and clinical evaluations conducted 24–72 h post-admission and at a 3-month follow-up.

Demographic, clinical, and blood test variables were collected for each patient, along with the time from symptom onset to neuroimaging, occlusion site, type of revascularization treatment, and the degree of final recanalization. The clinical severity of the stroke was addressed using the NIHSS, and the functional outcome at three months was addressed using the modified Rankin scale (mRS). As an observational cohort study, this work adheres to the STROBE guidelines [38].

**Study outcomes: definition of malignant cerebral infarction (MCI)**

The principal outcome, MCI, was defined through a combination of clinical and neuroimaging criteria [3,6,16,39]:

1. Clinical indicators of intracranial hypertension, such as a decreased level of consciousness (score ≥1 in the corresponding item on NIHSS), anisocoria, death due to cerebral edema, or need for decompressive craniectomy.
2. Neuroimaging evidence indicating significant cerebral edema, exemplified by a midline shift ≥6 mm or an infarct encompassing over half of the MCA territory.

A diagnosis of malignant stroke required the presence of at least one clinical and one neuroimaging criterion.

**Neuroimaging analysis**

The imaging protocol included a baseline multimodal whole-brain CT scan (total acquisition time, 83 s), which included NCCT (140 kV, 127 mAs, FOV = 225 mm, matrix = 512 × 512, section thickness = 5 mm); CTA (120 kV, 663 mAs, FOV = 261 mm, matrix = 512 × 512, section thickness = 0.6 mm); and CTP (80 kV[peak], 250 mAs, 1.5-s rotation, FOV = 18 mm, matrix = 512 × 512, and forty-nine 2-mm-thickness slices). Patients were scanned using a Somatom Definition Flash 128-section dual-source multidetector scanner (Siemens), with a 98-mm z-coverage and 26 time points acquired each 1.5 s and 4 last time points each 5 s (total acquisition time, 59 s). Fifty milliliters of nonionic iodinated contrast were administered intravenously at 5 mL/s using a power injector, followed by a saline flush of 20 mL at an injection rate of 2 mL/s.

The ASPECTS was assessed on the baseline NCCT. CTP maps were calculated by the commercial software MIStar 3.2 (Apollo Medical Imaging Technology, Melbourne, Australia) using a model-free singular-value decomposition algorithm with a delay and dispersion correction. The software automatically performs motion correction and selects an arterial input function from an unaffected artery (usually the anterior cerebral artery) and a venous output function from a large draining vein (the sagittal sinus). The software generates cerebral blood flow (CBF), cerebral blood volume (CBV), mean transient time (MTT), and delay time (DT) maps. Of note, the delay-corrected deconvolution method produces delay time maps rather than the more commonly used time-to-maximum (Tmax) maps [40]. A threshold of 3 s on the delay time maps was used to define the hypoperfusion [41], and the ischemic core was defined within the hypoperfused area with a series of relative CBF (rCBF) and CBV (rCBV) thresholds as a percentage of the mean perfusion values from the entire unaffected, contralateral hemisphere [42]. Vessel occlusion was assessed on CTA.

At 24 +/− 12 (median (IQR) 28 (18–33)) h, patients underwent an MRI study using a 3-T Prisma Siemens MR unit with a 32-channel phase-array coil, including 3-dimensional (3D) T1-weighted (T1w) imaging, DWI, dynamic susceptibility-weighted contrast-enhanced PWI, 3D time of flight angiography, susceptibility-weighted imaging, and fluid-attenuated inversion recovery. The final DWI lesion was segmented using Amira software (Visage Imaging, Berlin, Germany) [43]. Brain edema was quantified in FLAIR and segmented using the same software as in DWI. PWI maps were processed with Olea Sphere 3.0–SP6 (Olea Medical, Cambridge, MA, USA) [44]. The PWI deficits were quantified on Tmax within visually segmented areas of delayed perfusion to reduce artifacts, as previously reported [35]. To exclude small areas with increased Tmax due to noise, only patients with a minimum cluster size of >10 contiguous areas were quantified. The follow-up NCCT was performed on the same machine as the admission NCCT.

**Laboratory analysis**

Blood samples were collected from each patient upon admission and within the first 24 h after the initial evaluation. Complete blood counts were assessed with an automated hemocytometer. Each sample was immediately centrifuged and initially frozen at −20 °C and subsequently frozen at −80 °C for long-term storage. s100b, NSE, VEGF, and ICAM-1 were measured at a median delay of 20.5 h after baseline CTP (IQR 15.6–24.2 h) using commercial immunoassay kits and repeated from stored samples in cases of technical errors. The median delay from blood draw to follow-up MRI was 8.5 h.

**Statistical analysis**

Categorical variables were assessed using the Chi-square (χ2) test when appropriate, with Fisher’s exact test as an alternative for small sample sizes. Continuous variables following a normal distribution were presented using means and standard deviations (SD), whereas those not normally distributed, along with ordinal variables, were described using medians and interquartile ranges (IQR). Group comparisons for means and medians were conducted via the Student’s *t*-test, ANOVA, or Mann-Whitney U test, contingent upon the data’s distribution. Significance was set at *p* < 0.05 for all tests, and hypotheses were bilateral.

Malignant infarction prediction was modelled with stratified five-fold cross-validated logistic regression in three steps: (A) baseline clinical plus imaging variables (NIHSS at admission, ASPECTS, CTP rCBV < 30% volume); (B) plus treatment variables (rtPA use, mTICI and NIHSS posttreatment); (C) plus biomarkers (S100B at day 1, neutrophils, and glucose at admission). For infarct and FLAIR edema volumes we fit linear models and summarize fit with cross-validated R^2^; classification metrics (AUC, accuracy, precision, recall, F1) are reported for malignant infarction.

Analyses were performed with IBM SPSS v26, JASP v0.19.3, and Python 3.11 with pandas 2.2, numpy 1.26, scikitlearn 1.4, imbalancedlearn 0.12, statsmodels 0.14, SciPy 1.11, and figures were created with matplotlib 3.8.

## 3. Results

Out of seventy-three patients included, eighteen (24%) developed MCI. MCI patients were more frequently male (84 vs. 46%, *p* = 0.003) and younger (64 vs. 78 years old, *p* = 0.005) than non-MCI patients. As shown in Table 1, the remaining demographic and pre-stroke clinical characteristics were comparable between both groups, as was the symptom-onset to imaging interval.

Patients with MCI exhibited lower baseline ASPECTS (6 vs. 8, *p* = 0.006), higher infarct volume (105 vs. 66 mL, *p* = 0.010), more brain edema (182 vs. 78 mL, *p* < 0.001), and higher persistent hypoperfusion at the follow-up MRI (82 vs. 25 mL, *p* = 0.025). The cerebral perfusion profile on admission measured by CTP was also worse in MCI patients, with higher values of flow (57 vs. 25 mL, *p* = 0.005) and volume drop (31 vs. 11 mL, *p* < 0.001) compared to the contralateral cerebral hemisphere. MCI patients had higher blood glucose (164 vs. 133 mg/dL, *p* = 0.046 on admission; 143 vs. 119 mg/dL, *p* = 0.038 at 24 h), leukocyte (12.250 vs. 10.143 counts per 10^9^/L, *p* = 0.025 on admission; 14.646 vs. 10.282 counts per 10^9^/L, *p* < 0.001 at 24 h) and neutrophil counts (10.239 vs. 7.952 counts per 10^9^/L, *p* = 0.016 on admission; 12.561 vs. 8.461 counts per 10^9^/L, *p* < 0.001 at 24 h) on admission and at 24 h, as well as higher s100b (2.375 vs. 0.869 µg/mL, *p* = 0.019) and neuron-specific enolase values (27.235 vs. 20.891 ng/mL, *p* = 0.030) measured at 24 h after admission. There was no correlation between MCI and the other selected biomarkers. Radiological and laboratory variables are shown in Table 2.

**Figure 2:**
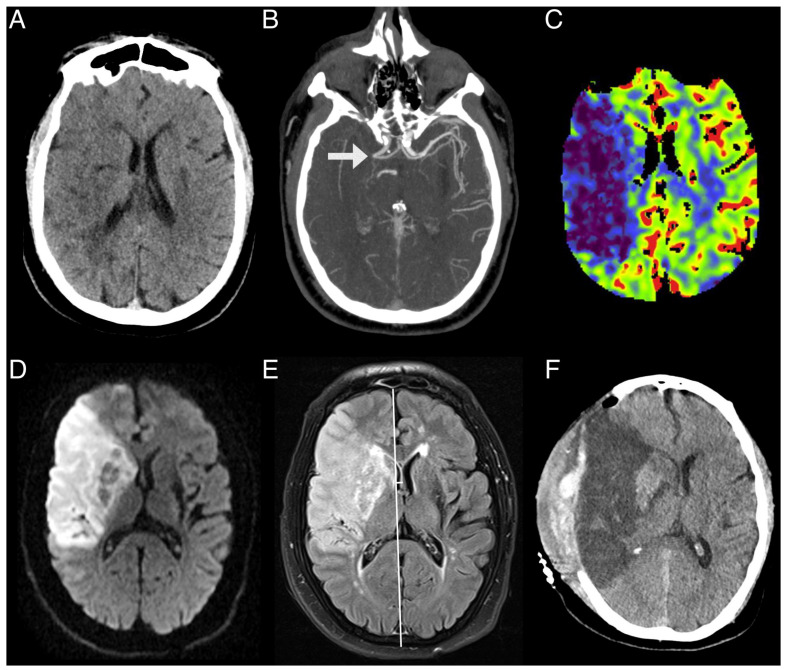
A previously healthy 55-year-old male transferred to the emergency department with severe right hemispheric syndrome (NIHSS: 20). NCCT (**A**) showed an extensive right ischemic lesion (ASPECTS 4) due to an occlusion of the M1 segment of the middle cerebral artery visualized on CTA ((**B**), arrow) and an extensive and severe volume drop on rCBV-based CTP, where the darker blue color indicates greater volume drops (**C**). Due to the extent of the injury, acute reperfusion treatment was not performed. The follow-up MRI performed 16 h after admission showed an extensive ischemic area on DWI (**D**), as well as abundant cerebral edema on FLAIR (**E**), causing a midline shift greater than 6 mm. A decompressive hemicraniectomy was performed 24 h after admission. The follow-up NCCT revealed an extensive ischemic lesion with cerebral edema and postsurgical changes (**F**). After the procedure, the patient survived but with serious sequelae that led to severe disability at 3 months.

Serum s100b concentration collected within the first 24 h showed a positive correlation with infarct volume, cerebral edema, and persistent hypoperfusion on MRI-PWI; but no correlation was observed between s100b and the initial perfusion profile measured in CTP. There was also no significant correlation between blood glucose levels, lymphocyte and neutrophil counts, NSE, ICAM1, and VEGF with infarct volume, cerebral edema, persistent hypoperfusion, and the amount of initial hypoperfusion. A paradigmatic example of the patients included in the study is shown in Figure 2.

Regarding functional outcome, patients with a successful functional outcome at 3 months had significantly lower values of s100b (0.28 vs. 0.67 µg/mL, *p* = 0.003). There were no significant associations between the remaining blood biomarkers and the functional outcomes.

**Figure 3:**
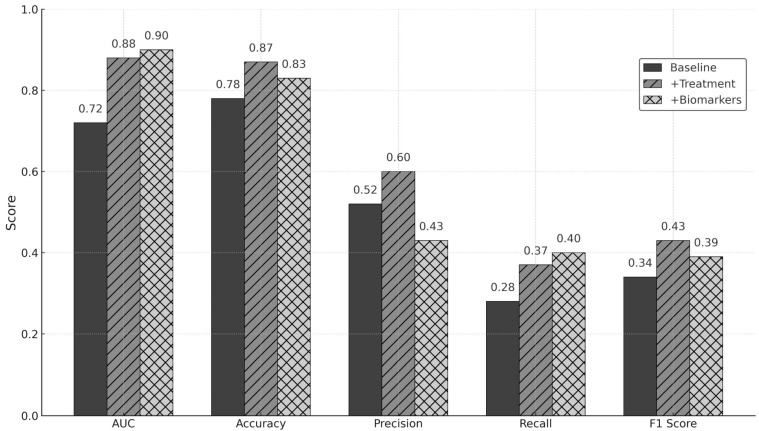
Performance metrics for malignant cerebral infarction by model set.

Figure 3 summarizes the predictive yield of the three predictive models. Across five-fold cross-validation, discrimination improved after adding treatment information and did not materially improve with biomarkers. The small AUC rise with biomarkers did not translate into better F1. Models based on baseline imaging and treatment-related variables were also the best at predicting infarct (R^2^ 0.27) and edema (R^2^ 0.58) volumes.

## 4. Discussion

In a cohort of patients with proximal LVO and severe neurological deficits, we found that clinical and radiological severity at admission yielded solid information for predicting the potential development of a malignant cerebral infarction. Thus, these findings support evidence from previous studies that describe the substantial predictive capacity of rCBV-based perfusion neuroimaging in identifying MCI [16]. Thus, although MRI is still considered the most accurate imaging test for diagnosing MCI [15,41], CTP, combined with clinical indicators of severity, offers a rapid and effective method for the early assessment of patients at risk for developing malignant cerebral edema with the additional benefit of being a widely available technique.

Regarding biomarkers, there is substantial literature that relates different elements to infarct volume, clinical severity, and prognosis. For example, s100b is an astroglial protein that has traditionally been studied in patients with different types of brain injuries, including ischemic stroke, and is related to the degree of injury and the functional outcome [20,31,45]. NSE is restricted to brain tissue and has been associated with ischemic brain damage [46,47], although with some discrepancies [33]. An association between VEGF and ICAM-1 and cerebral ischemia has been described [35,36,37]. Thus, we selected these markers specifically for their potential predictive value. However, although we also found significant correlations between serum levels of the s100b protein and the development of MCI, as well as the size of the infarct, cerebral edema, the degree of initial hypoperfusion, and persistent hypoperfusion, adding this information did not translate into improved prediction of MCI on top of the initial clinical and radiological information, and even less so when adding the presence or absence of successful recanalization at the end of the revascularization treatment: treatment informed models remained the most practical choice.

Thus, a simple approach using clinical data and widely available conventional neuroimaging is currently the most practical way to identify a patient profile that warrants comprehensive monitoring or other neuroimaging tests that would not otherwise be performed. While studying biomarkers could be valuable in developing neuroprotective agents for patients with severe ischemic stroke, we found that making predictive models more complex did not lead to improved clinical predictions.

Despite these results, other biomarkers could be of interest due to their involvement in the pathophysiological mechanisms that lead to the development of cerebral edema. For example, metalloproteases and interleukins involved in neuroinflammatory responses [48,49] could be related to the elevated leukocyte and neutrophil counts we observed in patients with MCI. Kisspeptin, on the other hand, is a neuropeptide that has recently been shown to be elevated in mouse models in cases of cerebral hemorrhage, particularly those associated with amyloid angiopathy [22], and that could play a role in restoring the permeability of the blood-brain barrier in cerebral ischemia [23]. For its part, the role of microRNA in ischemia-reperfusion injury, as well as in neuroprotection, has been described [25]. This, combined with the study of post-thrombectomy cerebral perfusion, could provide insight into the mechanisms of ischemic damage and potential neuroprotective therapies.

The strength of this study lies in its prospective and simple design, which was specifically focused on the study of predictive factors of MCI. Glucose or leukocyte populations are usually studied routinely when evaluating patients with LVO candidates for reperfusion treatments, hence making them ideal candidates for potential clinical application. The single determination of the additional biomarkers studied in a specific time frame prevented the assessment of time-dependent changes in their levels but also reflected better the use of biomarkers in real life. On the other hand, the fact that it is a single-center study may introduce limitations even if the design is easily reproducible. The number of patients included is relatively low but consistent with the described epidemiology. We deliberately focused on a narrowly defined, very severe phenotype. This increased the proportion of malignant evolution in our cohort (24%) relative to other stroke populations, but reduced heterogeneity and improved internal validity. The tradeoff is lower statistical power for subgroup analyses. Furthermore, the data were collected at a time when routine mechanical thrombectomy was not performed in patients with low ASPECTS. However, with the increasing inclination towards reperfusion therapies for extensive ischemic lesions, our findings could prove instrumental in identifying patients at increased risk for malignant cerebral edema after having received successful endovascular therapy.

The primary practical takeaway from our findings, focusing on the early identification of patients at risk for developing MCI, is that a collection of early clinical and radiological information can streamline decision-making and optimize resources, for example by prioritizing patient transfers to tertiary centers for close clinical monitoring with a view to potential surgical intervention.

## 5. Conclusions

Clinical severity and initial neuroimaging perfusion findings in patients with proximal LVO have solid value in early prediction of the development of MCI. Despite several associations between some circulating markers, such as the s100b protein, and clinical and radiological endpoints, blood biomarkers did not add significant predictive value on top of baseline information and response to treatment, which were the best predictors to identify patients at high risk of malignant conversion.

## Figures and Tables

**Table 1:** Clinical characteristics of the included patients according to the development of malignant MCA infarction.

	Total n = 73	Non-Malignant n = 55 (76%)	Malignant n = 18 (24%)	*p*-Value
**Female, n (%)**	33 (45)	30 (54)	3 (16)	0.003
**Age (years), median (IQR)**	76 (63–83)	78 (65–84)	64 (53–76)	0.005
**Premorbid mRS, median (IQR)**	0 (0–1)		0 (0–1)	0.961
**Hypertension, n (%)**	50 (68)	38 (69)	12 (66)	0.848
**Dyslipidemia, n (%)**	28 (38)	19 (34)	9 (50)	0.242
**Diabetes mellitus, n (%)**	17 (23)	11 (20)	6 (33)	0.245
**Smoker, n (%)**	16 (21)	12 (21)	4 (22)	0.971
**Alcohol intake (any dose), n (%)**	10 (13)	6 (10)	4 (22)	0.226
**Ischemic cardiopathy, n (%)**	11 (15)	6 (10)	5 (27)	0.082
**Atrial fibrillation, n (%)**	18 (24)	14 (25)	4 (22)	0.728
**Previous stroke, n (%)**	11 (15)	10 (18)	1 (5)	0.194
**Chronic kidney disease, n (%)**	10 (13)	6 (10)	4 (22)	0.226
**Occlusion site**				0.247
** M1, n (%)**	42 (57)	34 (61)	8 (44)	
** ICA, n (%)**	16 (21)	9 (16)	7 (38)	
** Tandem, n (%)**	16 (21)	12 (21)	4 (22)	
**NIHSS at admission, median (IQR)**	20 (17–24)	20 (17–24)	20 (18–22)	0.515
**NIHSS at 24 h, median (IQR)**	17 (13–22)	16 (11–20)	21 (17–33)	<0.001
**Treatment**				0.007
** None, n (%)**	14 (19)	8 (14)	6 (33)	
** rtPA, n (%)**	1 (1)	0 (0)	1 (5)	
** MT, n (%)**	39 (53)	28 (50)	11 (61)	
** MT + rtPA, n (%)**	19 (35)	19 (34)	0 (0)	
**mTICI**				0.188
** 0, n (%)**	17 (23)	9 (16)	8 (44)	
** 1, n (%)**	0 (0)	0 (0)	0 (0)	
** 2a, n (%)**	6 (8)	1 (1)	0 (0)	
** 2b, n (%)**	25 (34)	23 (41)	5 (27)	
** 2c, n (%)**	8 (10)	4 (7)	2 (11)	
** 3, n (%)**	37 (50)	18 (32)	4 (22)	
**Time-to-CTP in hours, mean (SD)**	7 (6)	7 (6)	8 (6)	0.571
**Systolic blood pressure (mmHg), mean (SD)**	144 (27)	145 (25)	143 (32)	0.828
**Hemorrhagic transformation**				
** None, n (%)**	24 (32)	22 (40)	2 (11)	
** HI1, n (%)**	5 (6)	4 (7)	1 (5)	
** HI2, n (%)**	13 (17)	9 (16)	4 (22)	
** PH1, n (%)**	16 (21)	14 (25)	2 (11)	
** PH2, n (%)**	4 (5)	3 (5)	1 (5)	
** SAH, n (%)**	6 (8)	1 (1)	5 (27)	
**Symptomatic hemorrhagic transformation, n%**	6 (8)	2 (3)	4 (22)	0.005
**TOAST**				0.320
** LAA, n (%)**	16 (21)	12 (21)	4 (22)	
** Cardioembolism, n (%)**	34 (46)	29 (52)	5 (27)	
** Undetermined, n (%)**	14 (19)	9 (16)	5 (27)	
** Other, n (%)**	8 (10)	5 (9)	3 (15)	
**mRS at 90 days, median (IQR)**	4 (3–6)	4 (2–5)	6 (5–6)	0.050

IQR: interquartile range; mRS: modified Rankin scale; M1: M1 segment of the middle cerebral artery; ICA: internal carotid artery; NIHSS: National Institute of Health Stroke Scale; rtPA: recombinant tissue plasminogen activator; MT: mechanical thrombectomy; mTICI: modified thrombolysis in cerebral infarction scale; CTP: computed tomography perfusion; mmHg: millimeters of mercury; SD: standard deviation; HI: hemorrhagic infarction; PH: parenchymal hematoma; SAH: subarachnoidal hemorrhage; TOAST: Trial of Org 10172 in Acute Stroke Treatment; LAA: large-artery atherosclerosis.

**Table 2:** Radiological and laboratory characteristics of the included patients according to the development of malignant MCA infarction.

	Total n = 73	Non-Malignant n = 55 (76%)	Malignant n = 18 (24%)	*p*-Value
**Infarct volume (DWI-MRI) in mL, mean (SD)**	73 (57)	66 (56)	105 (48)	0.010
**Brain edema (FLAIR-MRI) in mL, mean (SD)**	100 (70)	78 (52)	182 (73)	<0.001
**Persistent hypoperfusion (PWI-MRI) in mL, mean (SD)**	37 (58)	26 (46)	82 (82)	0.025
**ASPECTS, median (IQR)**	7 (5–9)	8 (6–9)	6 (3–8)	0.006
**rCBF < 30% (CTP) in mL, mean (SD)**	36 (36)	29 (28)	57 (49)	0.005
**rCBV < 30% (CTP) in mL, mean (SD)**	16 (21)	11 (14)	31 (30)	<0.001
**Basal glycaemia (mg/dL), mean (SD)**	141 (40)	133 (28)	164 (61)	0.046
**Glycaemia at 24 h (mg/dL), mean (SD)**	125 (41)	119 (36)	143 (50)	0.038
**Leucocytes at admission (counts per 10^9^/L), mean (SD)**	10.685 (3.471)	10.164 (3.411)	12.250 (3.255)	0.025
**Leucocytes at 24 h (counts per 10^9^/L), mean (SD)**	11.358 (3.819)	10.282 (2.782)	14.646 (4.689)	<0.001
**Neutrophils at admission** **(counts per 10^9^/L)** **, mean (SD)**	8.524 (3.623)	7.952 (3.554)	10.239 (3.359)	0.016
**Neutrophils at 24 h (counts per 10^9^/L), mean (SD)**	9.438 (2.804)	8.416 (2.943)	12.561 (4.480)	<0.001
**Lymphocytes at admission (counts per 10^9^/L), mean (SD)**	1.446 (0.766)	1.508 (0.785)	1.261 (0.692)	0.214
**Lymphocytes at 24 h (counts per 10^9^/L), mean (SD)**	1.360 (1.580)	1.415 (1.796)	1.192 (0.542)	0.985
**Platelets at admission (counts per 10^9^/L), mean (SD)**	219 (73)	226 (65)	199 (92)	0.188
**Platelets at 24 h** **(counts per 10^9^/L)** **, mean (SD)**	216 (79)	218 (67)	211 (109)	0.763
**s100b at 24 h (µg/mL), mean (SD)**	1.256 (2.431)	0.869 (1.130)	2.375 (4.284)	0.019
**NSE at 24 h (ng/mL), mean (SD)**	22.635 (14.462)	20.891 (9.668)	27.352 (22.696)	0.030
**VEGF at 24 h (pg/mL), mean (SD)**	115 (159)	115 (172)	114 (118)	0.986
**ICAM-1 at 24 h (ng/mL), mean (SD)**	165,170 (69,072)	160,883 (64,062)	177,578 (82,574)	0.367

SD: Standard Deviation; DWI-MRI: Diffusion-Weighted Imaging—Magnetic Resonance Imaging; FLAIR-MRI: Fluid Attenuated Inversion Recovery—Magnetic Resonance Imaging; PWI-MRI: Perfusion-Weighted Imaging—Magnetic Resonance Imaging; ASPECTS: Alberta Stroke Programme Early Computed Tomography Score; IQR: Interquartile Range; CTP: Computed Tomography Perfusion; rCBF: relative Cerebral Blood Flow; rCVB: relative Cerebral Blood Volume; µg/mL: micrograms per milliliter; NSE: Neuron Specific Enolase; ng/mL: nanograms per milliliter; VEGF: Vascular Endothelial Growth Factor; pg/mL: picograms per milliliter; ICAM-1: Intercellular Adhesion Molecule 1.

## Data Availability

Datasets produced and analyzed during this study can be obtained from the corresponding author upon a reasonable request.

## Abbreviations

The following abbreviations are used in this manuscript:

MCI	Malignant Cerebral Infarction
LVO	Large Vessel Occlusion
CT	Computed Tomography
CTP	Computed Tomography Perfusion
rCVB	Relative Cerebral Blood Volume
MRI	Magnetic Resonance Imaging
NSE	Neuron Specific Enolase
VEGF	Vascular Endothelial Growth Factor
ICAM1	Intercellular Adhesion Molecule 1
NIHSS	National Institute of Health Stroke Scale
ASPECS	Alberta Stroke Program Early Computed Tomography Score
rtPA	Recombinant Tissue Plasminogen Activator
mTICI	Modified Treatment in Cerebral Ischemia
AUC	Area Under Curve
MCA	Middle Cerebral Artery
NCCT	Non-Contrast Computed Tomography
CTA	Computed Tomography Angiography
mRS	Modified Rankin Score
rCBF	Cerebral Blood Flow
MTT	Mean Transient Time
DT	Delay Time
Tmax	Time-To-Maximum
DWI	Diffusion-Weighted Imaging
FLAIR	Fluid Attenuation Inversion Recovery
PWI	Perfusion-Weighted Imaging
